# Efficacy and safety of CD30-targeted chimeric antigen receptor T-cell therapy for lymphoma: a meta-analysis

**DOI:** 10.1186/s12885-026-16121-z

**Published:** 2026-05-25

**Authors:** Jixiang Shi, Cong Lu, Chao Song, Qian Wang

**Affiliations:** 1 Department of Hematology, ZiBo Central Hospital, No. 10 Shanghai Road, Zhangdian District, Zibo, Shandong 255000 China; 2 Department of Pathology, ZiBo Central Hospital, Zibo, 255000 China

**Keywords:** CD30-targeted chimeric antigen receptor T-cell therapy, Lymphoma, Efficacy, Safety, Meta-analysis

## Abstract

**Background:**

The clinical benefits of cluster of differentiation 30 (CD30)-target chimeric antigen receptor T-cell (CAR-T) therapy in patients with lymphoma remain controversial. This meta-analysis intended to comprehensively investigate the efficacy and safety of CD30-targeted CAR-T therapy in these patients.

**Methods:**

Studies on CD30-targeted CAR-T therapy for patients with lymphoma were searched comprehensively in Web of Science, PubMed, Embase, and Cochrane Library databases until March 2025. A single-arm meta-analysis was performed to calculate pooled proportions with 95% confidence intervals (CI). Heterogeneity was assessed using the I² statistic, and fixed- or random-effects models were applied accordingly. Efficacy and safety outcomes were extracted and analyzed.

**Results:**

A total of seven studies containing 102 cases were included in this meta-analysis. The pooled rates (95% CI) of complete response, partial response, stable disease, and progressive disease were 43.5% (17.6%; 69.3%), 17.5% (3.0%; 31.9%), 17.8% (3.0%; 32.6%), 10.7% (4.8%; 16.6%), respectively. The pooled objective response rate (95% CI) and disease control rate (95% CI) were 66.1% (46.4%; 85.9%) and 89.3% (83.4%; 95.2%). Regarding safety results, the pooled incidences (95% CI) of cytokine release syndrome, nausea or vomiting, anemia, and thrombocytopenia were 57.7% (37.7%; 77.8%), 38.2% (11.3%; 65.1%), 79.1% (48.0%; 100.0%), and 70.2% (36.1%; 100.0%), respectively. No publication bias was observed in any outcome measures (all *P* > 0.05). Quality assessment showed that all included studies were of moderate-to-high quality. Moreover, sensitivity analyses exhibited high robustness of results.

**Conclusion:**

CD30-targeted CAR-T therapy demonstrates promising objective response rates with manageable toxicities in patients with lymphoma. The findings of this meta-analysis provide preliminary descriptive evidence to inform future clinical research and exploratory application of CD30-targeted CAR-T therapy in lymphoma.

**Supplementary Information:**

The online version contains supplementary material available at 10.1186/s12885-026-16121-z.

## Introduction

Lymphoma is a heterogeneous group of diseases characterized by the clonal proliferation of lymphocytes, including Hodgkin lymphoma (HL) and non-Hodgkin lymphoma (NHL) [1, 2]. In recent years, the incidence of lymphoma has gradually increased, which brings a huge burden to the global medical system [3–5]. The treatment methods of lymphoma vary according to disease subtypes [1]. The standard treatment of HL is doxorubicin, bleomycin, vinblastine, and dacarbazine (ABVD) or bleomycin, etoposide, doxorubicin, cyclophosphamide, vincristine, procarbazine, and prednisone (BEACOPP) with or without radiation therapy [6]. Regarding NHL, it is usually treated with cyclophosphamide, doxorubicin, vincristine, and prednisone (CHOP) with or without rituximab (R-CHOP) [7]. Although most patients could be cured with the current therapies, a proportion of patients still develop relapsed or refractory disease, and their prognosis is unfavorable [8, 9]. Therefore, it is important to explore more treatment strategies to improve the management of lymphoma.

Chimeric antigen receptor T-cell (CAR-T) therapy uses engineered T cells to recognize tumor cell surface antigens and kill tumor cells, which has gradually been applied in treating lymphomas [10, 11]. Cluster of differentiation 30 (CD30) is a member of the tumor necrosis factor receptor family, which is overexpressed in multiple types of lymphoma [12, 13]. Some studies have reported that CD30-targeted CAR-T therapy achieved favorable treatment response with well-tolerated toxicity in treating patients with lymphoma [14–17]. However, in a recent study, it was suggested that a novel anti-CD30 CAR-T therapy (designated 5F11-28Z) showed a low treatment efficacy and significant hematologic toxicity in patients with lymphoma [18]. The above studies indicated that the clinical application of CD30-targeted CAR-T therapy in treating lymphoma is still controversial [14–18]. Thus, a comprehensive analysis is required for further verification.

Therefore, this meta-analysis reviewed data from available studies, intending to systematically investigate the efficacy and safety of CD30-targeted CAR-T therapy in patients with lymphoma.

## Methods

### Literature search and eligible criteria

The Web of Science, PubMed, Embase, and Cochrane Library databases were searched comprehensively to find relevant studies on CD30-targeted CAR-T therapy for patients with lymphoma. The search strategy included the following keywords: “CD30”, “CD30^+^”, “anti-CD30”, “CD30-targeting”, “CD30-directed”, “CD30 antigen”, “CAR-T”, “chimeric antigen receptor T-cell”, “lymphoma”, “lymphomas”, and “lymphocytes”. The search was last updated in March 2025. The full search strategies for all databases were shown in Supplementary Table 1. This meta-analysis was conducted and reported in accordance with the Preferred Reporting Items for Systematic Reviews and Meta-Analyses (PRISMA) guidelines.

The criteria for study inclusion were: (1) investigated CD30-targeted CAR-T therapy in patients with lymphoma; (2) reported clinical outcomes of therapy (efficacy or safety); and (3) published in English. The exclusion criteria were: (1) case reports, meta-analyses, reviews, or experiments (cells or animal studies); (2) studies from the same research group with a high overlap of patient populations; or (3) conference abstracts without sufficient data. Two reviewers independently performed the search, and discrepancies were resolved by discussion. This meta-analysis was not pre-registered, as the study protocol was developed after the initiation of the literature search.

### Data extraction and quality assessment

Information on study characteristics, patient demographics, dosage of CAR-T cells, and clinical outcomes were collected. The outcome measures included efficacy and safety evaluation of therapy. The efficacy outcomes comprised complete response (CR), partial response (PR), stable disease (SD), progressive disease (PD), objective response rate (ORR), and disease control rate (DCR). The safety outcomes included cytokine release syndrome (CRS), nausea or vomiting, anemia, and thrombocytopenia. Due to the recommendation of at least 3 articles for the publication bias test, a meta-analysis was conducted for indexes reported in 3 or more studies in this study. The methodological index for non-randomized studies (MINORS) method was used to assess the quality of included studies [19]. The certainty of evidence for each outcome was assessed using the Grading of Recommendations Assessment, Development and Evaluation (GRADE) approach. Two reviewers independently performed the assessment, and discrepancies were resolved by discussion.

### Statistical analysis

R version 4.3.2 was used for statistical analyses. A meta-analysis of single-arm studies was performed, and pooled estimates were calculated as single-group proportions with a 95% confidence interval (CI). Before pooling, the normality test of event rates was conducted via the Shapiro-Wilk test. If the *P* value of the normality test was over 0.05, the data were considered normally distributed, and no transformation was required. In this study, all models met the normality assumption (*P* > 0.05).

Heterogeneity among studies was estimated via the *I²* and Cochran’s Q tests. A random effects model was applied if significant heterogeneity (*I²*>50% or *P* < 0.10) was present; otherwise, a fixed effects model was performed. Both Peters’ and Begg’s tests were performed to assess publication bias. The stability of the model was assessed by sensitivity analysis. Subgroup analyses on study region were conducted to explore the sources of heterogeneity. Due to data limitations, a descriptive summary of efficacy and safety outcomes according to lymphoma subtypes was performed.

## Results

### Study screening process

**Fig. 1:**
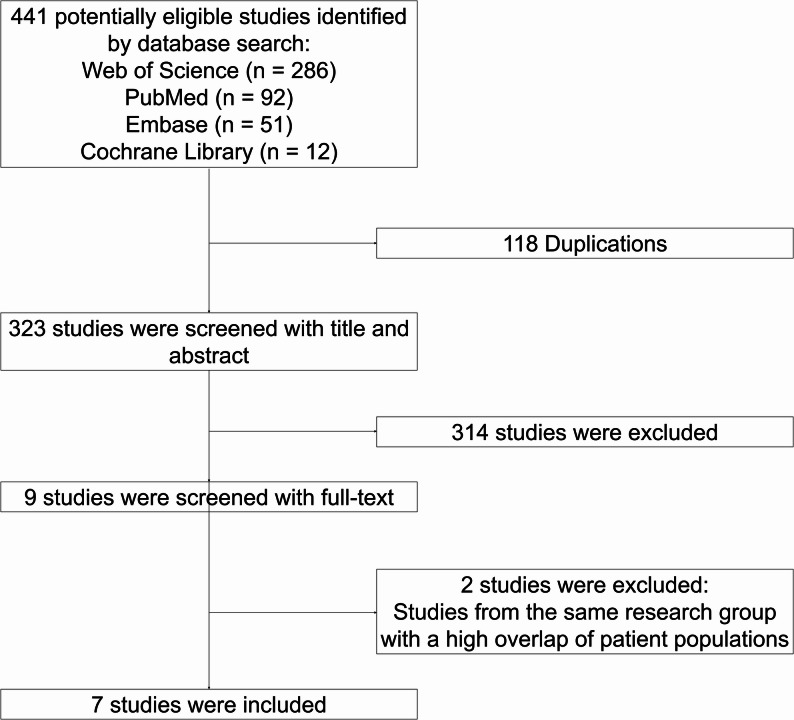
Study processing flow

In total, 441 potentially eligible studies were identified by database search, including 286 from Web of Science, 92 from PubMed, 51 from Embase, and 12 from Cochrane Library. A total of 118 duplicated studies were excluded. After screening with title and abstract, 314 studies were removed. Then, 9 studies were screened with full-text reading, and 3 studies were from the same research group with a high overlap of patient populations. To avoid duplication, 2 of the 3 studies were excluded. Ultimately, 7 studies were included in this meta-analysis [14–18, 20, 21] (Fig. 1).

### Information on included studies

The 7 studies were published between 2017 and 2024, with a total sample size of 102 cases, including 68 males and 34 females. All studies were single-armed. There were 4 studies conducted in China and 3 studies conducted in the USA. More specific characteristics of included studies, such as lymphoma type, the dosage of CAR-T cells, and analysis indexes, are exhibited in Table 1. In addition, key baseline characteristics of the included patients, including lines of previous treatment, relapsed/refractory status, ECOG performance status, and tumor burden, are summarized in Supplementary Table 2.

**Table 1:** Characteristics of analyzed studies

Study ID (First author, publication year)	Country	Study type	Sample size	Age range (years)	Male/Female sex	Lymphoma type	Dosage of CAR-T cells	Analysis indexes
Ramos CA, 2017 [20]	USA	Phase I, single-arm	9	20.0–65.0	6/3	r/r HL and ALCL	0.2-2.0*10^8^ /m^2^	CR, PR, SD, PD, ORR, DCR
Wang CM, 2017 [14]	China	Phase I, single-arm	18	13.0–77.0	13/5	ALCL and HL	Mean: 1.56*10^7^/kg	CR, PR, SD, PD, ORR, DCR, Nausea or vomiting
Wang D, 2020 [15]	China	Pilot study, single-arm	9	20.0–46.0	5/4	ALCL and HL	0.7–3.2*10^7^ /kg	CR, PR, SD, PD, ORR, DCR, CRS, Thrombocytopenia, Anemia, Nausea or vomiting
Sang W, 2022 [16]	China	Phase II, single-arm	12	19.0–64.0	7/5	cHL, AITL, and GZL	0.1-1.0*10^7^ /kg	CR, PR, SD, PD, ORR, DCR, CRS, Nausea or vomiting
Voorhees TJ, 2022 [21]	USA	Phase I/II, single-arm	27	15.0–67.0	18/9	r/r cHL	1.0–2.0*10^8^ /m^2^	CR, PR, SD, PD, ORR, DCR
Zhang P, 2022 [17]	China	Pilot study, single-arm	6	18.0–30.0	4/2	cHL and ALCL	Median: 7.6*10^6^ /kg	CR, PR, SD, PD, ORR, DCR, CRS, Thrombocytopenia, Anemia, Nausea or vomiting
Brudno JN, 2024 [18]	USA	Phase I, single-arm	21	18.0–64.0	15/6	cHL	0.3-9.0*10^6^ /kg	CR, PR, SD, PD, ORR, DCR, CRS, Thrombocytopenia, Anemia

*CAR* Chimeric antigen receptor, *r/r* Relapsed/refractory, *HL* Hodgkin lymphoma, *ALCL* Anaplastic large cell lymphoma, *CR* Complete response, *PR* Partial response, *SD* Stable disease, *PD* Progressive disease, *ORR* Objective response rate, *DCR* Disease control rate, *CRS* Cytokine release syndrome, *cHL* Classical Hodgkin lymphoma, *AITL* Angioimmunoblastic T-cell lymphoma, *GZL* Gray zone lymphoma

### Quality assessment

The MINORS tool was used to assess the quality of the 7 studies included in this meta-analysis. With a total score of 16, the MINORS score was 9 for 1 study, 11 for 5 studies, and 12 for 1 study, indicating a moderate-to-high quality of these studies (Table 2).

**Table 2:** Quality assessment by MINORS tool

Study ID	1st item	2nd item	3rd item	4th item	5th item	6th item	7th item	8th item	Total MINORS score
Ramos CA, 2017	2	0	2	2	2	1	0	0	9
Wang CM, 2017	2	1	2	2	2	1	1	0	11
Wang D, 2020	2	1	2	2	2	1	1	0	11
Sang W, 2022	2	1	2	2	2	1	1	0	11
Voorhees TJ, 2022	2	1	2	2	2	2	1	0	12
Zhang P, 2022	2	1	2	2	2	2	0	0	11
Brudno JN, 2024	2	1	2	2	2	1	1	0	11

MINORS, Methodological index for non-randomized studies

1st item: A clearly stated aim

2nd item: Inclusion of consecutive patients

3rd item: Prospective collection of data

4th item: Endpoints appropriate to the aim of the study

5th item: Unbiased assessment of the study endpoint

6th item: Follow-up period appropriate to the aim of the study

7th item: Loss to follow-up less than 5%

8th item: Prospective calculation of the study size

The total score ranged from 0 to 16. For each item, 0 represented data not reported, 1 represented data reported but inadequate, and 2 represented reported and adequate

### Treatment efficacy evaluation

**Fig. 2:**
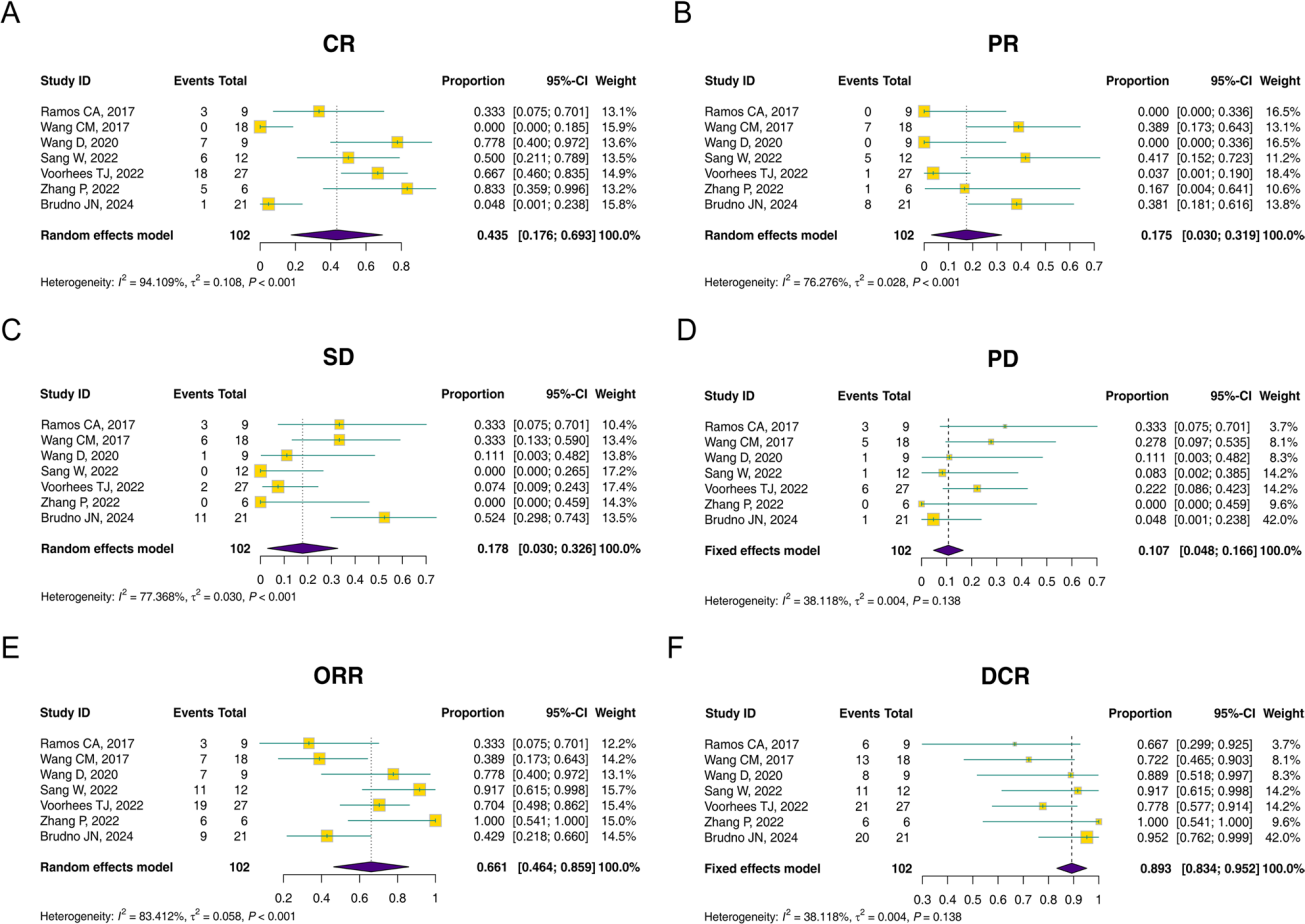
Forest plots of the treatment efficacy. The CR (**A**), PR (**B**), SD (**C**), PD (**D**), ORR (**E**), and DCR (**F**) in patients with lymphoma who received CD30-targeted CAR-T therapy

The seven studies assessed CR, PR, SD, PD, ORR, and DCR in patients with lymphoma who received CD30-targeted CAR-T therapy. There was heterogeneity in the CR rate among these studies (*I*^2^ = 94.109%, *P* < 0.001). The random effects model illustrated that the CR rate (95% CI) was 43.5% (17.6%; 69.3%) (Fig. 2A). The heterogeneity also existed in the PR rate among studies (*I*^2^ = 76.276%, *P* < 0.001), and the random effects model showed that the PR rate (95% CI) was 17.5% (3.0%; 31.9%) (Fig. 2B). The heterogeneity in the SD rate was observed among studies (*I*^2^ = 77.368%, *P* < 0.001), and the SD rate (95% CI) evaluated by the random effects model was 17.8% (3.0%; 32.6%) (Fig. 2C). No heterogeneity was found in the PD rate among these studies (*I*^2^ = 38.118%, *P* = 0.138), and the fixed effects model revealed that the PD rate (95% CI) was 10.7% (4.8%; 16.6%) (Fig. 2D). Regarding ORR, heterogeneity was observed among the 7 studies (*I*^2^ = 83.412%, *P* < 0.001), and the random effects model revealed that ORR (95% CI) was 66.1% (46.4%; 85.9%) (Fig. 2E). There was no heterogeneity in DCR among studies (*I*^2^ = 38.118%, *P* = 0.138). The fixed effects model illustrated that DCR (95% CI) was 89.3% (83.4%; 95.2%) (Fig. 2F).

### Safety evaluation

**Fig. 3:**
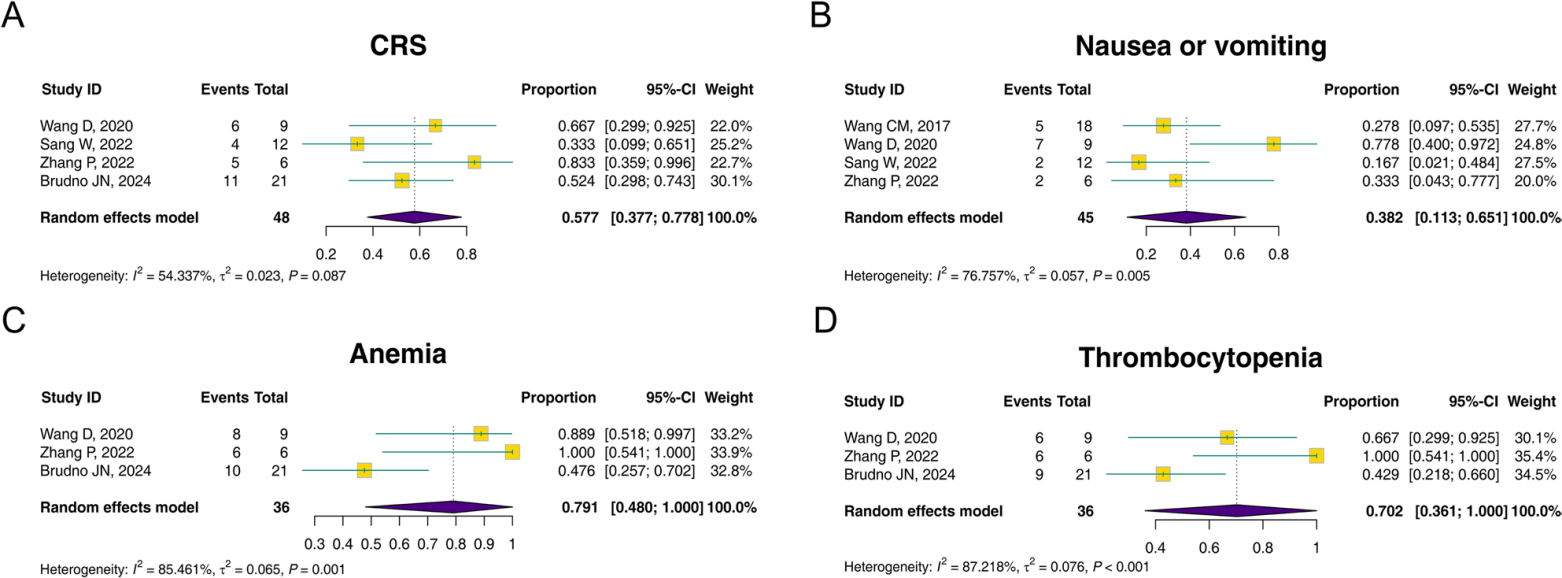
Forest plots of adverse reactions. The incidences of CRS (**A**), nausea or vomiting (**B**), anemia (**C**), and thrombocytopenia (**D**) in patients with lymphoma who received CD30-targeted CAR-T therapy

Four studies assessed the incidence of CRS in patients with lymphoma who received CD30-targeted CAR-T therapy, and there was heterogeneity (*I*^2^ = 54.337%, *P* = 0.087). The random effects model disclosed the incidence (95% CI) of CRS was 57.7% (37.7%; 77.8%) (Fig. 3A). There was heterogeneity among four studies that evaluated the incidence of nausea or vomiting (*I*^2^ = 76.757%, *P* = 0.005). The random effects model found that the incidence (95% CI) of nausea or vomiting was 38.2% (11.3%; 65.1%) (Fig. 3B). Three studies assessed the incidence of anemia, and heterogeneity was observed (*I*^2^ = 85.461%, *P* = 0.001). The random effects model showed that the incidence (95% CI) of anemia was 79.1% (48.0%; 100.0%) (Fig. 3C). Heterogeneity also existed among three studies that assessed the incidence of thrombocytopenia (*I*^2^ = 87.218%, *P* < 0.001). The random effects model illustrated that the incidence (95% CI) of thrombocytopenia was 70.2% (36.1%; 100.0%) (Fig. 3D). It should be noted that the upper limits of the 95% CI for anemia and thrombocytopenia reached 100.0%, which may be attributed to the small sample size and high heterogeneity across studies, reflecting statistical uncertainty rather than true incidence. In addition to the pooled safety outcomes, other adverse events were reported in these studies. A descriptive summary was provided in the Supplementary Table 3. Overall, these findings suggested that, in addition to the commonly reported adverse events included in the meta-analysis, CD30-targeted CAR-T therapy may be associated with a broad spectrum of hematological, metabolic, and neurological toxicities, highlighting the need for comprehensive monitoring and supportive management in clinical practice.

### Subgroup analysis

A descriptive summary of key efficacy and safety outcomes stratified by lymphoma subtype was presented in Supplementary Table 4. Subgroup analyses based on study region (USA vs. China) showed that there were no significant differences in most efficacy outcomes (CR, PR, SD, and ORR) or CRS incidence (all *P* for subgroup difference > 0.05). However, significant differences were observed for hematological toxicities, including anemia (*P* < 0.001) and thrombocytopenia (*P* = 0.030), with higher incidences reported in studies from China. Detailed results are presented in Supplementary Table 5. The heterogeneity observed across studies may be partly explained by differences in lymphoma subtypes and study characteristics.

Publication bias and sensitivity analyses.

By Peters’ test, there was no publication bias in CR, PR, SD, PD, ORR, DCR, the incidence of CRS, or the incidence of nausea or vomiting (all *P* > 0.05). Moreover, no publication bias was observed in CR, PR, SD, PD, ORR, DCR, the incidences of CRS, nausea or vomiting, anemia, or thrombocytopenia by Begg’s test (all *P* > 0.05) (Table 3).

**Table 3:** Publication bias

Items	*P* value (Peters’ test)	*P* value (Beggs’ test)
CR	0.939	0.652
PR	0.769	0.219
SD	0.737	0.099
PD	0.791	0.099
ORR	0.912	0.293
DCR	0.791	0.099
CRS	0.456	0.497
Nausea or vomiting	0.657	0.497
Anemia	(-)	0.117
Thrombocytopenia	(-)	0.602

*SE* Standard error, *CR* Complete response, *PR* Partial response, *SD* Stable disease, *PD* Progressive disease, *ORR* Objective response rate, *DCR* Disease control rate, *CRS* Cytokine release syndrome

Special statement: The notation “(-)” indicated that the *P* value could not be calculated by Peters’ test because there were extreme values in the analysis data. As a result, after deleting the studies where the extreme values were located, the number of studies was less than 3, and the *P* value could not be calculated

In terms of sensitivity analyses, the results, including CR, PR, SD, PD, ORR, DCR, the incidences of CRS, nausea or vomiting, anemia, or thrombocytopenia, would not be affected by removing each study, indicating the high robustness of the results (Table 4). The certainty of evidence for all outcomes, as assessed by the GRADE approach, is summarized in Supplementary Table 6. The overall evidence level ranged from low to moderate. Most outcomes, including CR, PR, SD, ORR and main safety indicators, were rated as low certainty.

**Table 4:** Sensitivity analyses

Items	Proportion [95% CI]
CR	
Omitting Ramos CA, 2017	0.453 [0.153; 0.752]
Omitting Wang CM, 2017	0.514 [0.261; 0.767]
Omitting Wang D, 2020	0.380 [0.106; 0.654]
Omitting Sang W, 2022	0.427 [0.126; 0.728]
Omitting Voorhees TJ, 2022	0.395 [0.105; 0.686]
Omitting Zhang P, 2022	0.372 [0.107; 0.638]
Omitting Brudno JN, 2024	0.505 [0.239; 0.772]
Pooled estimate	0.435 [0.176; 0.693]
PR	
Omitting Ramos CA, 2017	0.210 [0.050; 0.370]
Omitting Wang CM, 2017	0.139 [0.000; 0.285]
Omitting Wang D, 2020	0.210 [0.050; 0.370]
Omitting Sang W, 2022	0.142 [0.000; 0.287]
Omitting Voorhees TJ, 2022	0.207 [0.041; 0.374]
Omitting Zhang P, 2022	0.179 [0.015; 0.344]
Omitting Brudno JN, 2024	0.138 [0.000; 0.284]
Pooled estimate	0.175 [0.030; 0.319]
SD	
Omitting Ramos CA, 2017	0.161 [0.000; 0.323]
Omitting Wang CM, 2017	0.155 [0.000; 0.318]
Omitting Wang D, 2020	0.192 [0.018; 0.367]
Omitting Sang W, 2022	0.215 [0.053; 0.378]
Omitting Voorhees TJ, 2022	0.203 [0.027; 0.378]
Omitting Zhang P, 2022	0.209 [0.045; 0.373]
Omitting Brudno JN, 2024	0.106 [0.003; 0.209]
Pooled estimate	0.178 [0.000; 0.326]
PD	
Omitting Ramos CA, 2017	0.099 [0.039; 0.159]
Omitting Wang CM, 2017	0.092 [0.031; 0.154]
Omitting Wang D, 2020	0.107 [0.045; 0.169]
Omitting Sang W, 2022	0.111 [0.048; 0.175]
Omitting Voorhees TJ, 2022	0.088 [0.025; 0.152]
Omitting Zhang P, 2022	0.119 [0.057; 0.181]
Omitting Brudno JN, 2024	0.151 [0.073; 0.228]
Pooled estimate	0.107 [0.048; 0.166]
ORR	
Omitting Ramos CA, 2017	0.708 [0.506; 0.909]
Omitting Wang CM, 2017	0.707 [0.501; 0.913]
Omitting Wang D, 2020	0.642 [0.414; 0.870]
Omitting Sang W, 2022	0.614 [0.402; 0.826]
Omitting Voorhees TJ, 2022	0.652 [0.416; 0.887]
Omitting Zhang P, 2022	0.604 [0.411; 0.798]
Omitting Brudno JN, 2024	0.701 [0.487; 0.914]
Pooled estimate	0.661 [0.464; 0.859]
DCR	
Omitting Ramos CA, 2017	0.901 [0.841; 0.961]
Omitting Wang CM, 2017	0.908 [0.846; 0.969]
Omitting Wang D, 2020	0.893 [0.831; 0.955]
Omitting Sang W, 2022	0.889 [0.825; 0.952]
Omitting Voorhees TJ, 2022	0.912 [0.848; 0.975]
Omitting Zhang P, 2022	0.881 [0.819; 0.943]
Omitting Brudno JN, 2024	0.850 [0.772; 0.927]
Pooled estimate	0.893 [0.834; 0.952]
CRS	
Omitting Wang D, 2020	0.555 [0.286; 0.824]
Omitting Sang W, 2022	0.651 [0.464; 0.838]
Omitting Zhang P, 2022	0.499 [0.337; 0.662]
Omitting Brudno JN, 2024	0.604 [0.309; 0.899]
Pooled estimate	0.577 [0.377; 0.778]
Nausea or vomiting	
Omitting Wang CM, 2017	0.424 [0.050; 0.798]
Omitting Wang D, 2020	0.238 [0.100; 0.375]
Omitting Sang W, 2022	0.464 [0.142; 0.786]
Omitting Zhang P, 2022	0.398 [0.039; 0.757]
Pooled estimate	0.382 [0.113; 0.651]
Anemia	
Omitting Wang D, 2020	0.740 [0.227; 1.000]
Omitting Zhang P, 2022	0.684 [0.279; 1.000]
Omitting Brudno JN, 2024	0.949 [0.809; 1.000]
Pooled estimate	0.791 [0.480; 1.000]
Thrombocytopenia	
Omitting Wang D, 2020	0.716 [0.156; 1.000]
Omitting Zhang P, 2022	0.520 [0.293; 0.747]
Omitting Brudno JN, 2024	0.856 [0.533; 1.000]
Pooled estimate	0.703 [0.361; 1.000]

*CI* Confidence interval, *CR* Complete response, *PR* Partial response, *SD* Stable disease, *PD* Progressive disease, *ORR* Objective response rate, *DCR* Disease control rate, *CRS* Cytokine release syndrome

## Discussion

CAR-T cell therapy is an immunotherapy that has brought clinical benefits for a variety of human malignancies, including lymphoma [22, 23]. At present, CD19-targeted CAR-T therapy has been approved as an effective treatment strategy for patients with lymphoma [24]. A previous study found that CD19-targeted CAR-T therapy yielded an ORR of 63.0% and a DCR of 84.0% in patients with relapsed/refractory large B-cell lymphoma [25]. Another study also disclosed that the ORR and DCR were 68.4% and 84.2% in patients with relapsed/refractory diffuse large B-cell lymphoma who received CD19-targeted CAR-T therapy [26]. Similar to CD19, CD30 also serves as a promising target in treating lymphoma, while its clinical efficacy is still being explored [13, 27, 28]. Herein, this meta-analysis comprehensively explored the treatment response of CD30-targeted CAR-T therapy in patients with lymphoma. The results showed that the ORR and DCR were 66.1% and 89.3%, respectively. The ORR and DCR observed in this study appear to be within a similar range to those reported in previous studies of CD19-targeted CAR-T therapy [25, 26]. However, such comparisons should be interpreted cautiously, as differences in study design, patient populations, and treatment settings preclude direct comparison between these therapies. Therefore, the findings should be considered as preliminary descriptive evidence rather than indicative of comparable efficacy between CD30-targeted and CD19-targeted CAR-T therapies. In addition, the differences in treatment response may also be partly explained by the biological characteristics of CD30 expression across lymphoma subtypes. In detail, CD30 is known to be strongly and uniformly expressed in certain subtypes, such as anaplastic large cell lymphoma, which may enhance the recognition and killing activity of CD30-targeted CAR-T cells [29]. In contrast, classical Hodgkin lymphoma is characterized by a unique tumor microenvironment, including abundant immunosuppressive cells and cytokines, which may affect CAR-T cell function [30]. In addition, other lymphoma subtypes may show more variable CD30 expression, which could further contribute to differences in therapeutic efficacy. These biological differences may partly account for the variability in response rates observed across studies and highlight the importance of considering subtype-specific characteristics when interpreting the results.

Although CAR-T cell therapy showed promising efficacy, its toxicity cannot be ignored [31, 32]. A previous study found that CRS, neurological events, and immune effector cell-associated neurotoxicity syndrome (ICANS) occurred in 48.5%, 37.1%, and 4.1% of patients with relapsed/refractory B-cell lymphoma who received CD19-targeted CAR-T cell therapy [33]. In another study, the incidence of nausea or vomiting was 35.2% in patients with lymphoma who received CAR-T therapy [34]. In addition, a study showed that the incidences of anemia, thrombocytopenia, and neutropenia were 51.0%, 80.0%, and 94.0% in patients treated with CD19-targeted CAR-T cell therapy [35]. This meta-analysis illustrated that the incidences of CRS, nausea or vomiting, anemia, and thrombocytopenia were 57.7%, 38.2%, 79.1%, and 70.2%, respectively, in patients with lymphoma who received CD30-targeted CAR-T therapy. The incidence of anemia in our meta-analysis was different from that in previous studies, while the incidences of CRS, nausea or vomiting, and thrombocytopenia were similar [33–35]. Overall, these adverse events appeared to be generally manageable with appropriate supportive care; however, the safety profile should be interpreted cautiously due to the limited sample size and single-arm design. In addition, some of the included studies also reported other adverse events, including neutropenia, purpura, rash, leukopenia, fatigue, diarrhea, and hyperuricemia [14–18]. These adverse events were not included in this meta-analysis, which was because: (1) The incidence of neutropenia was 100% in patients with lymphoma who received CD30-targeted CAR-T therapy in three included studies [15, 17, 18]. (2) The number of studies reporting the incidences of purpura, rash, leukopenia, fatigue, diarrhea, and hyperuricemia was less than three [14–18]. However, given the single-arm design and limited sample size of the included studies, these findings should be interpreted cautiously and cannot be used to establish definitive clinical recommendations. Furthermore, the relatively high incidences of anemia and thrombocytopenia observed in this study also have important clinical implications. In clinical practice, these adverse events are generally manageable with appropriate supportive care, including close monitoring, blood transfusion, etc. Therefore, early identification and appropriate management of hematological toxicities are essential to ensure the safety of patients receiving CD30-targeted CAR-T therapy.

The baseline characteristics of the included patients should be taken into consideration when interpreting the results of this study. Most patients were heavily pretreated and were in relapsed or refractory status, with multiple prior lines of therapy. In addition, although ECOG performance status was generally ≤ 2 in the reported studies, the tumor burden showed obvious variation, and many patients presented with advanced-stage disease or extranodal involvement. These baseline features may have an influence on both the efficacy and safety outcomes of CD30-targeted CAR-T therapy. For example, patients with relapsed or refractory disease and multiple prior treatments may have more aggressive disease and impaired immune condition, which could affect the expansion and persistence of CAR-T cells. Therefore, the pooled results of this study may mainly reflect the outcomes in a high-risk population, and caution is needed when extending these findings to patients with less prior treatment or earlier-stage disease. Based on this, CD30-targeted CAR-T therapy may have potential clinical benefit in patients with relapsed or refractory CD30-positive lymphoma, particularly those who have failed multiple lines of prior treatment. Besides, it may serve as a salvage therapeutic option for patients with limited treatment alternatives. However, further studies are still needed to verify these findings.

MINORS method is a valid tool used to evaluate the quality of studies, which contains 12 items and the first 8 items are specifically for non-comparative studies [19]. Due to the fact that all included studies were single-armed, this meta-analysis used the first 8 items of MINORS for quality assessment. The scores ranged from 9 to 12, indicating a moderate-to-high quality of these studies. Notably, all included studies scored 0 regarding the prospective calculation of the study size. This might be because: Lymphoma is uncommon and it is difficult to collect a large number of patients, thus the prospective calculation of the study size was not performed. There was no publication bias in any analysis indexes, whether conducted by Peters’ test or Begg’s test. Moreover, sensitivity analyses exhibited a high robustness of results.

The heterogeneity observed across studies may be partly attributable to differences in lymphoma subtypes. The subgroup analysis based on study region suggested that geographic factors may partially contribute to differences in hematological toxicities, including the anemia and thrombocytopenia. However, these findings should be interpreted cautiously due to the small number of studies and persistent heterogeneity within subgroups. In addition, variations in CAR-T cell dosing strategies across studies may represent another important source of heterogeneity. Differences in dose levels, infusion schedules, and CAR-T cell constructs could influence both therapeutic efficacy and toxicity profiles. However, due to inconsistent reporting formats, the impact of CAR-T dose could not be quantitatively assessed in the present analysis. Furthermore, heterogeneity may also arise from differences in patient baseline characteristics, including disease status (relapsed/refractory), prior lines of therapy, tumor burden, and performance status, as well as variations in study design and follow-up duration. These factors may collectively affect treatment response and adverse event incidence, thereby contributing to the observed variability across studies. Therefore, the pooled estimates should be interpreted as overall trends across heterogeneous populations rather than subtype-specific effects, which may limit the direct clinical extrapolation of these findings.

Several limitations should be noted: (1) Some important clinical outcome measures could not be included in this meta-analysis due to insufficient reporting. (2) Only single-arm studies were included, while no controlled trials were included, resulting in a relatively low level of evidence and interpretation of the causal relationship. (3) In addition, this study was not pre-registered, which may increase the risk of potential bias. (4) There was heterogeneity in lymphoma subtypes in these studies, which may affect the interpretation of pooled results. (5) The dosage of CAR-T cells varied considerably among studies, which may contribute to heterogeneity in both efficacy and safety outcomes. (6) The overall sample size was relatively small, which may affect the generalizability of the findings.

## Conclusion

In conclusion, CD30-targeted CAR-T therapy demonstrates potential treatment benefit with generally manageable toxicity profiles in patients with lymphoma. However, these findings should be interpreted cautiously given the lack of control groups and the low level of evidence, and are intended to inform future research rather than guide clinical decision-making.

## Supplementary Information

Supplementary Material 1.

Supplementary Material 2.

Supplementary Material 3.

Supplementary Material 4.

Supplementary Material 5.

Supplementary Material 6.

Supplementary Material 7.

## Data Availability

The datasets generated or analyzed during the study are available from the corresponding author on reasonable request.

## Abbreviations

CD30: Cluster of differentiation 30
CAR-T: Chimeric antigen receptor T-cell
CI: Confidence interval
HL: Hodgkin lymphoma
NHL: Non-Hodgkin lymphoma
ABVD: doxorubicin, bleomycin, vinblastine, and dacarbazine
BEACOPP: Bleomycin, etoposide, doxorubicin, cyclophosphamide, vincristine, procarbazine, and prednisone
CHOP: Cyclophosphamide, doxorubicin, vincristine, and prednisone
R-CHOP: Rituximab cyclophosphamide, doxorubicin, vincristine, and prednisone
MINORS: Methodological index for non-randomized studies
CR: Complete response
PR: Partial response
SD: Stable disease
PD: Progressive disease
ORR: Objective response rate
DCR: Disease control rate
