# Supplementary material for: Efficacy and safety of CD30-targeted chimeric antigen receptor T-cell therapy for lymphoma: a meta-analysis
Source: BMC Cancer. 2026 May 25;26:876. doi: 10.1186/s12885-026-16121-z (PMC13386688; doi:10.1186/s12885-026-16121-z)
Supplement: Supplementary file 1 — Supplementary Material 1. [file 12885_2026_16121_MOESM1_ESM.docx]

**Supplementary Table 1.** The detailed search strategies for each database.

| Database | Search strategies |
| --- | --- |
| Web of Science | ((TS=(CD30) OR TS=(anti-CD30) OR TS=(CD30 antigen)) AND TS=(chimeric antigen receptor T-cell)) AND TS=(lymphoma) |
| PubMed | ("CD30"[All Fields] OR "CD30+"[All Fields] OR "anti-CD30"[All Fields] OR "CD30-targeting"[All Fields] OR "CD30-directed"[All Fields] OR "CD30 antigen"[All Fields]) AND ("chimeric antigen receptor T-cell"[All Fields] OR "CAR-T"[All Fields]) AND ("lymphoma"[All Fields] OR "lymphomas"[All Fields] OR "lymphocytes"[All Fields]) |
| Embase | ('CD30'):ti,ab,kw AND (('chimeric antigen receptor T-cell'):ti,ab,kw) AND (('lymphoma'):ti,ab,kw) |
| Cochrane Library | #1 CD30  #2 anti-CD30  #3 chimeric antigen receptor T-cell  #4 lymphoma  #5 #1 OR #2  #6 #5 AND #3 AND #4 |
