# Supplementary material for: Efficacy and safety of CD30-targeted chimeric antigen receptor T-cell therapy for lymphoma: a meta-analysis
Source: BMC Cancer. 2026 May 25;26:876. doi: 10.1186/s12885-026-16121-z (PMC13386688; doi:10.1186/s12885-026-16121-z)
Supplement: Supplementary file 2 — Supplementary Material 2. [file 12885_2026_16121_MOESM2_ESM.docx]

**Supplementary Table 2.** Supplementary information on characteristics of analyzed studies.

| Study ID  (First author, publication year) | Lines of previous treatment | r/r status | ECOG PS | Tumor burden |
| --- | --- | --- | --- | --- |
| Ramos CA, 2017 [20] | ≥3 | Reported as r/r | Not reported | Not reported |
| Wang CM, 2017 [14] | >10 | Partially reported as r/r | 2 or less | Extensive abnormal lymph node regions (range, 0-7); 83% with extranodal disease |
| Wang D, 2020 [15] | ≥2 | Reported as r/r | 2 or less | 5 with stage II; 1 with stage III; 3 with stage IV |
| Sang W, 2022 [16] | ≥1 | Reported as r/r | 2 or less | 9 with extranodal invasion |
| Voorhees TJ, 2022 [21] | Median of 8 (range,  3-23) | Reported as r/r | Not reported | 9 with I-II stage; 18 with III-IV stage |
| Zhang P, 2022 [17] | ≥2 | Reported as r/r | Not reported | 2 with II stage; 2 with III stage; 2 with IV stage |
| Brudno JN, 2024 [18] | Median of 7 (range,  4-15) | Partially reported as relapsed | 2 or less | Median metabolic tumor volume of 66.1 mL (range, 6.4-486.7 mL) |

r/r, relapsed/refractory; ECOG PS, Eastern Cooperative Oncology Group performance status.
