# Supplementary material for: Efficacy and safety of CD30-targeted chimeric antigen receptor T-cell therapy for lymphoma: a meta-analysis
Source: BMC Cancer. 2026 May 25;26:876. doi: 10.1186/s12885-026-16121-z (PMC13386688; doi:10.1186/s12885-026-16121-z)
Supplement: Supplementary file 3 — Supplementary Material 3. [file 12885_2026_16121_MOESM3_ESM.docx]

**Supplementary Table 3.** Characteristics of analyzed studies.

| Study ID  (First author, publication year) | Adverse events |
| --- | --- |
| Ramos CA, 2017 [20] | None of the adverse events reported (most frequently fatigue, hyper- or hypokalemia, and transient elevation of AST) were considered related to CD30. No patients developed symptoms consistent with cytokine release syndrome. |
| Wang CM, 2017 [14] | Breathlessness; Psychiatric abnormalities; Urticaria; Joint swelling; Dizziness; Pneumonitis (lung infection); Triglyceride increase; Uric acid increase; Alanine aminotransferase increase; Aspartate aminotransferase increase; γ-GGT increase |
| Wang D, 2020 [15] | Purpura; Cytopenias; Neutropenia; Leukopenia; Hyperuricemia; Transaminitis; Hypoalbuminemia |
| Sang W, 2022 [16] | Rash; Cytopenias; Fatigue; Diarrhea |
| Voorhees TJ, 2022 [21] | None |
| Zhang P, 2022 [17] | Purpura; Hypotension; Oral mucositis; Diarrhea; Prolonged Activated Partial Thromboplastin Time; Hyperuricemia; Transaminitis; Hypoalbuminemia |
| Brudno JN, 2024 [18] | Rash; Neutropenia; Dizziness; Confusion; Headache; Hallucinations |
