# Supplementary material for: Efficacy and safety of CD30-targeted chimeric antigen receptor T-cell therapy for lymphoma: a meta-analysis
Source: BMC Cancer. 2026 May 25;26:876. doi: 10.1186/s12885-026-16121-z (PMC13386688; doi:10.1186/s12885-026-16121-z)
Supplement: Supplementary file 4 — Supplementary Material 4. [file 12885_2026_16121_MOESM4_ESM.docx]

**Supplementary Table 4.** Descriptive summary of key efficacy and safety outcomes according to lymphoma subtypes across included studies.

| Study ID  (First author, publication year) | Lymphoma type | CR (%) | PR (%) | ORR (%) | DCR (%) | CRS (%) | Anemia (%) | Thrombocytopenia (%) |
| --- | --- | --- | --- | --- | --- | --- | --- | --- |
| Ramos CA, 2017 | r/r HL and ALCL | 33.3 | 0.0 | 33.3 | 66.7 | NR | NR | NR |
| Wang CM, 2017 | ALCL and HL | 0.0 | 38.9 | 38.9 | 72.2 | NR | NR | NR |
| Wang D, 2020 | ALCL and HL | 77.8 | 0.0 | 77.8 | 88.9 | 66.7 | 88.9 | 66.7 |
| Sang W, 2022 | cHL, AITL, and GZL | 50.0 | 41.7 | 91.7 | 91.7 | 33.3 | NR | NR |
| Voorhees TJ, 2022 | r/r cHL | 66.7 | 3.7 | 70.4 | 77.8 | NR | NR | NR |
| Zhang P, 2022 | cHL and ALCL | 83.3 | 16.7 | 100 | 100 | 83.3 | 100 | 100 |
| Brudno JN, 2024 | cHL | 4.8 | 38.1 | 42.9 | 95.2 | 52.4 | 47.6 | 42.9 |

CR, complete response; PR, partial response; ORR, objective response rate; DCR, disease control rate; CRS, cytokine release syndrome; HL, Hodgkin lymphoma; ALCL, anaplastic large cell lymphoma; NR, not report
