# Supplementary material for: Efficacy and safety of CD30-targeted chimeric antigen receptor T-cell therapy for lymphoma: a meta-analysis
Source: BMC Cancer. 2026 May 25;26:876. doi: 10.1186/s12885-026-16121-z (PMC13386688; doi:10.1186/s12885-026-16121-z)
Supplement: Supplementary file 5 — Supplementary Material 5. [file 12885_2026_16121_MOESM5_ESM.docx]

**Supplementary Table 5.** Subgroup analysis of high-heterogeneity efficacy and safety outcomes by study region.

| Outcomes/Subgroup | No. of studies | Pooled estimate (95% CI) | *I^2^* | *P* for subgroup difference |
| --- | --- | --- | --- | --- |
| **CR** |  |  |  | 0.545 |
| USA | 3 | 0.344 [0.000; 0.714] | 94.711% |  |
| China | 4 | 0.510 [0.119; 0.902] | 95.168% |  |
| **PR** |  |  |  | 0.508 |
| USA | 3 | 0.124 [0.000; 0.343] | 80.635% |  |
| China | 4 | 0.227 [0.015; 0.438] | 76.156% |  |
| **SD** |  |  |  | 0.212 |
| USA | 3 | 0.295 [0.015; 0.574] | 86.875% |  |
| China | 4 | 0.095 [0.000; 0.239] | 62.190% |  |
| **ORR** |  |  |  | 0.134 |
| USA | 3 | 0.511 [0.287; 0.736] | 67.861% |  |
| China | 4 | 0.777 [0.511; 1.000] | 84.257% |  |
| **CRS** |  |  |  | 0.665 |
| USA | 1 | 0.524 [0.298; 0.743] | (-) |  |
| China | 3 | 0.604 [0.309; 0.899] | 68.519% |  |
| **Nausea or vomiting** |  |  |  | (-) |
| USA | 0 | (-) | (-) |  |
| China | 4 | 0.382 [0.113; 0.651] | 76.757% |  |
| **Anemia** |  |  |  | <0.001 |
| USA | 1 | 0.476 [0.257; 0.702] | (-) |  |
| China | 2 | 0.949 [0.809; 1.000] | 0.000% |  |
| **Thrombocytopenia** |  |  |  | 0.030 |
| USA | 1 | 0.429 [0.218; 0.660] | (-) |  |
| China | 2 | 0.856 [0.533; 1.000] | 69.250% |  |

CI, confidence interval; CR, complete response; PR, partial response; SD, stable disease; ORR, objective response rate; CRS, cytokine release syndrome.
