# Supplementary material for: Efficacy and safety of CD30-targeted chimeric antigen receptor T-cell therapy for lymphoma: a meta-analysis
Source: BMC Cancer. 2026 May 25;26:876. doi: 10.1186/s12885-026-16121-z (PMC13386688; doi:10.1186/s12885-026-16121-z)
Supplement: Supplementary file 6 — Supplementary Material 6. [file 12885_2026_16121_MOESM6_ESM.docx]

**Supplementary Table 6.** Evaluation of evidence certainty.

| Outcome | Risk of Bias | Inconsistency | Indirectness | Imprecision | Publication Bias | Certainty |
| --- | --- | --- | --- | --- | --- | --- |
| CR | Serious | Serious | Serious | Not serious | Undetected | Low |
| PR | Serious | Serious | Serious | Not serious | Undetected | Low |
| SD | Serious | Serious | Serious | Not serious | Undetected | Low |
| PD | Serious | Not serious | Serious | Not serious | Undetected | Moderate |
| ORR | Serious | Serious | Serious | Not serious | Undetected | Low |
| DCR | Serious | Not serious | Serious | Not serious | Undetected | Moderate |
| CRS | Serious | Not serious | Serious | Serious | Undetected | Low |
| Nausea or vomiting | Serious | Serious | Serious | Serious | Undetected | Low |
| Anemia | Serious | Serious | Serious | Serious | Undetected | Low |
| Thrombocytopenia | Serious | Serious | Serious | Serious | Undetected | Low |

The certainty of evidence was categorized as high, moderate, low, or very low.
